# Inter-site harmonization based on dual generative adversarial networks for diffusion tensor imaging: application to neonatal white matter development

**DOI:** 10.1186/s12938-020-0748-9

**Published:** 2020-01-15

**Authors:** Jie Zhong, Ying Wang, Jie Li, Xuetong Xue, Simin Liu, Miaomiao Wang, Xinbo Gao, Quan Wang, Jian Yang, Xianjun Li

**Affiliations:** 1 Department of Radiology, The First Affiliated Hospital of Xi’an Jiaotong University, Xi’an, 710061 China; 2 School of Electronic Engineering, Xidian University, Xi’an, 710071 China; 3 Key Laboratory of Biomedical Spectroscopy of Xi’an, Xi’an Institute of Optics and Precision Mechanics, Chinese Academy of Sciences, Xi’an, 710119 China

**Keywords:** Harmonization, Diffusion tensor imaging, Neonate, Generative adversarial network

## Abstract

**Background:**

Site-specific variations are challenges for pooling analyses in multi-center studies. This work aims to propose an inter-site harmonization method based on dual generative adversarial networks (GANs) for diffusion tensor imaging (DTI) derived metrics on neonatal brains.

**Results:**

DTI-derived metrics (fractional anisotropy, FA; mean diffusivity, MD) are obtained on age-matched neonates without magnetic resonance imaging (MRI) abnormalities: 42 neonates from site 1 and 42 neonates from site 2. Significant inter-site differences of FA can be observed. The proposed harmonization approach and three conventional methods (the global-wise scaling, the voxel-wise scaling, and the ComBat) are performed on DTI-derived metrics from two sites. During the tract-based spatial statistics, inter-site differences can be removed by the proposed dual GANs method, the voxel-wise scaling, and the ComBat. Among these methods, the proposed method holds the lowest median values in absolute errors and root mean square errors. During the pooling analysis of two sites, Pearson correlation coefficients between FA and the postmenstrual age after harmonization are larger than those before harmonization. The effect sizes (Cohen’s *d* between males and females) are also maintained by the harmonization procedure.

**Conclusions:**

The proposed dual GANs-based harmonization method is effective to harmonize neonatal DTI-derived metrics from different sites. Results in this study further suggest that the GANs-based harmonization is a feasible pre-processing method for pooling analyses in multi-center studies.

## Background

Diffusion tensor imaging (DTI) has been widely used to assess structural alterations associated with the brain development or lesions on neonates [1, 2]. However, the sample size is always limited due to the difficulty of the neonatal data acquisition [3]. To improve the statistical power, the multi-center/multi-scanner study is a common strategy [4]. DTI-derived metrics are reproductive when magnetic resonance imaging (MRI) scanners and acquisition protocols are equivalent [5]. However, differences related to the variety of scanners, magnetic fields, coils, and/or acquisition protocols usually exist in multi-center studies [6, 7]. Such site-specific effects will introduce measurement variability, which hinder the ability to obtain ‘truly’ quantitative measures, which may lead to false findings [8]. Therefore, the site-specific variations have to be removed prior to integrating datasets. The inter-site (or inter-scanner) harmonization is the essential step in multi-center studies.

Recently, several harmonization methods have been proposed based on the phantom or directly based on human brain datasets. The phantom-based harmonization is a simple and feasible approach to correct systematic differences across sites [4, 9]. However, it is not suitable for retrospective studies to monitor real-time states of MRI scanners by phantoms. Furthermore, it may be not adequate to capture tissue-specific differences [7]. The harmonization based on human brain datasets may overcome these problems related to the phantom-based harmonization [7, 10–13]. There are three categories of approaches based on human brain datasets [7, 11]: the model-free approach based on the rotation-invariant spherical harmonic (RISH) features, the meta-analysis, and the statistical covariates methods. For the RISH-based method, regional complexities of biological properties in the brain have been considered during the harmonization [10]. It is able to capture tissue-specific differences. However, the accuracy of the transformation from diffusion-weighted images to the representation of spherical harmonic basis depends on the gradient direction number [14]. The demand of the high angular resolution during the data acquisition will limit its clinical applications. Approaches based on DTI-derived metrics, such as fractional anisotropy (FA) and mean diffusivity (MD), may be relatively feasible in clinical applications. As a comparison, the harmonization based on the meta-analysis can be performed on DTI-derived metrics. The meta-analysis strategy [15, 16] harmonizes data through calculating z-scores, with the hypothesis of Gaussian distribution of the metric. However, the distribution is non-Gaussian because of the limited sample size in most cases [11]. Similarly, harmonization methods based on statistical covariates can also be performed on DTI-derived metrics. Among the methods based on statistical covariates, the linear scaling based on the whole brain or the target region of interest is the easiest to be implemented [4, 17]. Compared with the scaling, ComBat demonstrates better performances by estimating additive and multiplicative factors in each voxel [17, 18]. However, the parametric distribution is difficult to be determined for various imaging models [7]. It is also difficult to determine whether assumed parameters in the ComBat are enough to reflect scanner-related or site-related effects. Moreover, the feasibility of the previous harmonization methods proposed on datasets of adults remains to be investigated on datasets of neonates.

To solve the problem related to the parameter selection and capture characteristics of the data distribution, the framework of generative adversarial networks (GANs) is an effective approach [19, 20]. Moreover, deep learning-based algorithms can reliably capture the nonlinear mapping relationship between different sites or scanners [13]. GANs employ two neural networks, the generator and the discriminator, to yield high-quality synthetic images. By setting opposite objectives, the generator and the discriminator are adversarial to each other. As the training goes on, each network will be improved. Finally, the generator can yield sharp vivid images. Based on the power of GANs to generate synthetic images, the dual learning architecture has been adapted to achieve the unsupervised image-to-image translation [21–23]. This motivates us to consider the same demand in the harmonization task.

**Fig. 1:**
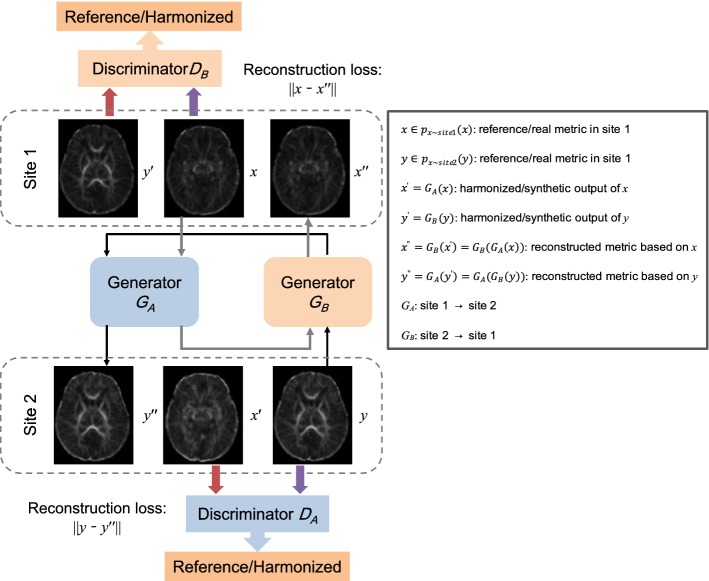
The diagram of the proposed harmonization approach based on dual generative adversarial networks

**Fig. 2:**
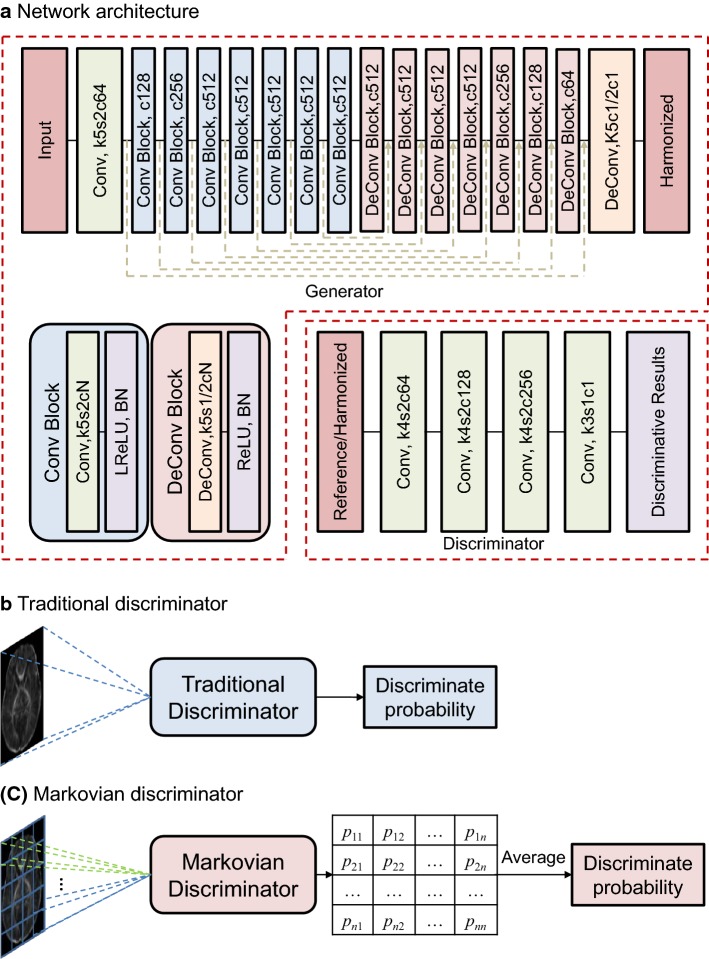
Network architecture (**a**) and differences between the Markovian and the traditional discriminators (**b**, **c**). *Conv* convolution operation, *DeConv* transposed convolution operation, *kN* kernel size, *sN* stride size, *cN* number of output channels, *BN* batch normalization layers, *ReLU* rectified linear unit, *LReLU*, Leaky rectified linear unit. The green dotted line in generator represents skip connection followed by concatenation operation. *p*_*ij*_ (*i* = 1,2,…,*n*; *j* = 1,2,…,*n*) denotes the discriminate probability computed in each local region, where *i* and *j* are the orders of regions in the horizontal and the vertical orientations

In this work, we try to propose an inter-site harmonization approach by using the dual GANs (Fig. 1) with the Markovian discriminator (Fig. 2). Based on DTI-derived metrics (FA and MD) of age-matched neonates from different sites, this study introduces the GANs to find the complex nonlinear mapping relationship between two different domains. Performances of the proposed approach are compared with three conventional methods: the scaling based on the whole brain white matter (global-wise scaling), the scaling in voxels (voxel-wise scaling), and ComBat [17]. The highlight of this work is to propose a dual GANs-based harmonization method and evaluate its performance on neonatal datasets.

## Results

### DTI metrics affected by sites

**Fig. 3:**
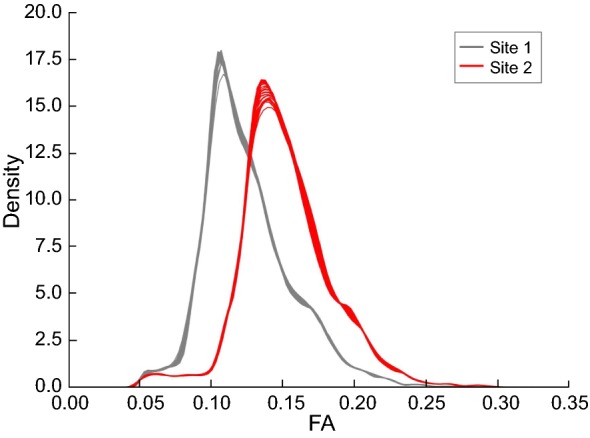
Distributions of fractional anisotropy (FA) values on white matter of datasets from different sites. Note that site 2 holds significantly (*P *< 0.05) higher FA than site 1

**Fig. 4:**
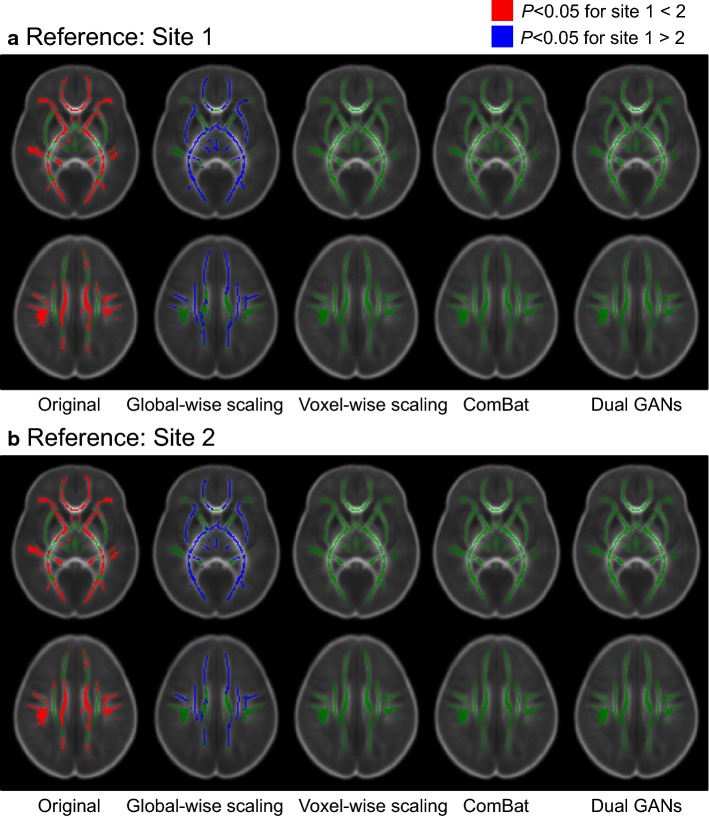
Inter-site differences in fractional anisotropy (FA) values before and after harmonization using different methods. Red represents that site 2 holds significantly (*P *< 0.05) higher FA than site 1. Blue represents that site 2 holds significantly (*P *< 0.05) lower FA than site 1. Green represents regions without significant difference between two sites. Note: dual generative adversarial networks are performed with the 2-dimensional kernel on axial slices. For inter-site comparisons after harmonization, differences with *P *< 0.05 in any run of the sixfold cross-validation are overlaid together on the template map

To demonstrate site effects on FA, Fig. 3 shows the histogram of FA values in the white matter region on neonates from two sites. In this study, there is no significant inter-site difference in the gestational age, the postmenstrual age, the gender ratio, or the birth weight of the enrolled neonates. However, site 2 holds significantly (*P *< 0.05) higher FA than site 1 (as shown in Fig. 3). Moreover, Pearson correlation coefficient between the inter-site difference and the averaged FA is 0.1771 (as shown in Figure S1, Additional file 1). This suggests that the correlation between FA values and site effects is weak. By using tract-based spatial statistics (TBSS), it can be found that the original data without harmonization have significant inter-site differences (*P *< 0.05) in nearly the whole white matter region (as shown in Fig. 4). Similar site effects can also be found on MD (Figure S2A and B, Additional file 2). Though the inter-site differences in the distribution of MD is less obvious than that of FA, TBSS shows significant differences in MD between sites. This is in agreement with the previous results on subject from 8 to 19 years [17]. Therefore, it is necessary to harmonize the data prior to the pooling analysis.

### Dual GANs reduce site-related effects on DTI-derived metrics

As shown in Fig. 4, the proposed method achieves comparable results as the voxel-wise scaling and the ComBat methods. They can eliminate the inter-site significant differences in the white matter region. However, quantities of voxels with inter-site significant differences still exist for the global-wise scaling method. This suggests that methods performed at the voxel level are more efficient than that performed at the global level.

**Fig. 5:**
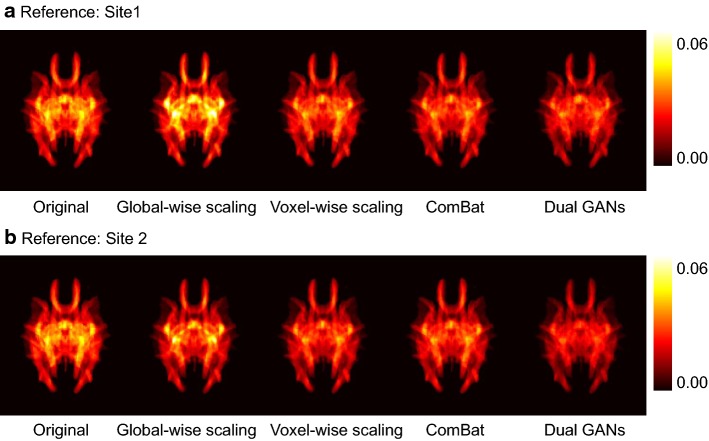
Representative slices of the averaged absolute error before and after harmonization by using different methods. Note: dual generative adversarial networks are performed with the 2-dimensional kernel on axial slices. Absolute errors after harmonization have been averaged over different runs of the sixfold cross-validation

**Fig. 6:**
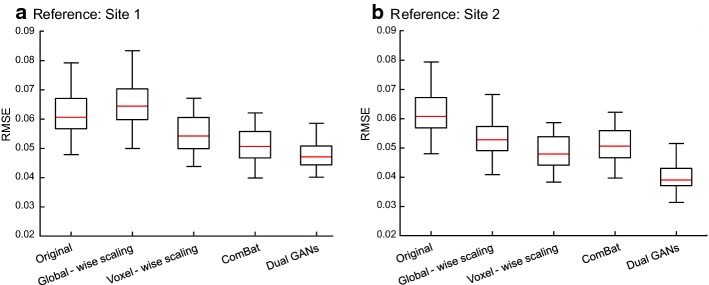
Boxplot of root mean square error (RMSE) on white matter before and after harmonization by using different methods. The red lines represent median values in RMSE. Note: dual generative adversarial networks are performed with the 2-dimensional kernel on axial slices. Prior to the calculation of medians and the boxplot drawing, RMSE values after harmonization have been averaged over different runs of the sixfold cross-validation

To quantify differences between sites, Figs. 5 and 6 show the absolute error and the root mean square error (RMSE) before and after harmonization. The voxel-wise scaling, the ComBat, and the proposed dual GANs methods can reduce the absolute error and RMSE, compared with those of the original data. Furthermore, the proposed method holds the lowest median values in the absolute error and RMSE, according to the results of the sixfold cross-validation.

Note that, the results in Figs. 4, 5, and 6 are based on the dual GANs with the 2-dimensional (2D) kernel in the axial orientation. Compared with the sagittal or the coronal orientation, the harmonization based on dual GANs with 2D-kernel in the axial orientation holds lower RMSE (as shown in Table S1, Additional file 3). It can achieve comparable results with the 3-dimensional (3D) kernel (as shown in Table S1, Additional file 3 and Figure S3, Additional file 4). Moreover, the harmonization based on dual GANs with the 2D-kernel in the axial orientation can remove site effects on MD as well (as shown in Figure S2, Additional file 2). As in the case of 3 sites, one of the sites can be selected as the reference. As shown in Figure S4, Additional file 5, site 2 is considered as the reference. The dual-GAN method can remove the differences across sites (Figure S4B and D, Additional file 5).

### Application in the pooling analysis of white matter development

**Fig. 7:**
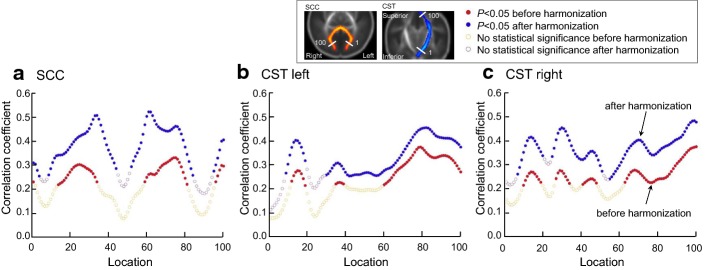
Correlation coefficient (Pearson correlation) between fractional anisotropy (FA) along white matter tracts and the postmenstrual age before and after harmonization. Locations: 100 planes along the white matter tract. Red and blue dots represent significant (*P *< 0.05) correlation before and after harmonization, respectively. Note: dual generative adversarial networks are performed with the 2-dimensional kernel on axial slices. Correlation coefficients after harmonization have been averaged over different runs of the sixfold cross-validation. The *P *≥ 0.05 in any run of the cross-validation indicates that the correlation is not statistically significant

To reveal age-related changes during the pooling analysis, this study performs Pearson correlation between postmenstrual age and FA along splenium of the corpus callosum (SCC), left and right corticospinal tract (CST) on neonates without MRI abnormalities. Positive correlation between the postmenstrual age and FA can be found (as shown in Fig. 7). The dual GANs harmonization method increases the number of locations with significant correlation (*P *< 0.05), compared with the correlation before harmonization. Moreover, correlation coefficients after harmonization averaged over different runs of the cross-validation are also larger than those before harmonization.

Furthermore, inter-gender differences (Cohen’s *d* between males and females) can be maintained by the harmonization procedure (as shown in Figure S5, Additional file 6). Specifically, changes in the Cohen’s *d* are from 0.1088 to 0.0990, from − 0.2177 to − 0.2632, and from − 0.3328 to − 0.4226 on SCC, left CST and right CST separately in site 1. As in site 2, changes in the Cohen’s *d* are from 0.2598 to 0.2343, from 0.1528 to 0.1141, and from 0.2894 to 0.2980 on SCC, left CST and right CST separately. Note that the Cohen’s d values after harmonization have been averaged over different runs of the cross-validation.

## Discussion

This study proposes a dual GANs-based harmonization method for neonatal DTI-derived metrics from different sites. With smaller errors than conventional methods, the proposed method effectively removes site-related effects during the TBSS analysis. Moreover, the method can preserve the age-related and the gender-related variations of FA during the harmonization procedure.

### Dual GANs vs. scaling and ComBat methods

Differences between sites or scanners, specifically including differences in magnetic fields, coils, and acquisition parameters, always cause nonlinear changes in MRI signals [7]. Together with potential unknown factors, the above differences make the relationship between two sites complex. To remove effects associated with sites or scanners, various studies have proposed several methods to harmonize the DTI data [4, 17]. Consistent with the previous finding [17], the global-wise scaling does not work well to harmonize neonatal datasets from different sites in this study. This is due to the spatial heterogeneity of site-related effects throughout the white matter [10]. As comparisons, the scaling and the ComBat at the voxel-wise level can overcome the problem of the global-wise scaling and perform well on neonatal datasets. However, it is not able to fully capture the nonlinear inter-site relationship by using a prior assumed model with several observing significant factors. Different from conventional methods like the scaling and the ComBat, the proposed method introduces dual GANs to map the complex nonlinear relationship between different sites. This relatively complex mapping procedure takes longer time than the scaling and ComBat methods. On the same local computer, the scaling and ComBat methods take about 2 × 10^−5^ s/slice, while the proposed method takes 2 × 10^−2^ s/slice during the inference procedure. Fortunately, the proposed method demonstrates advantages in reducing inter-site differences with smaller absolute errors and RMSE. The age-related and the gender-related variations of FA can be preserved during the harmonization procedure. Furthermore, the dual GANs-based harmonization can increase the correlation coefficient between FA and the postmenstrual age during the pooling analysis. In the case of multiple sites (more than 2 sites), one of the sites should be selected as the reference during the harmonization procedure. Results in this study suggest that dual GANs can be an alternative method for the data harmonization in multi-center studies.

### Dual GANs vs. other deep learning-based methods

Besides the assumed model-based harmonization methods, several deep learning approaches have also been reported [12, 13]. Similar to the motivation of those deep learning-based methods, this work tries to map the complex relationship between sites through the convolutional neural network. Different from previous methods, the proposed approach is based on DTI-derived metrics instead of RISH features, considering the limited gradient direction number [14]. Meanwhile, this work uses the Markovian discriminator in the harmonization framework. This can improve the ability of GANs to capture the local information [22, 23], though the local information may be still not enough to achieve details in site effects. Furthermore, the dual GANs-based harmonization can work well on unpaired datasets during the training, which will improve the flexibility during applications.

As for the selection of kernel styles of dual GANs, the 2D-kernel in the axial orientation shows great performances, compared with the 3D-kernel and 2D-kernels in the coronal and the sagittal orientations. This may be associated with the acquisition mode. In this current work, the axial acquisition mode is used. The intra-slice resolution is 1.41 × 1.41 mm^2^, while the slice thickness is 2.5 mm or 4 mm. Therefore, the harmonization depending on axial slices may be more suitable for these datasets. Considering the efficiency of training (2D vs. 3D: 0.07 vs. 0.70 s/slice) and the RMSE, this work focuses on the GANs with the 2D-kernel. During applications, the appropriate orientation should be selected according to the acquisition mode.

### Limitations

Despite the promising results, this study also has some limitations. Firstly, the proposed method should be used with caution during applications, though it works well on the enrolled neonates in this study. Model parameters in dual GANs are mainly dependent on the target and the reference images. Therefore, dual GANs should be trained again when they are performed on new datasets to capture the specific information. Secondly, the mapping between sites in this work may be not perfect. This work harmonizes DTI-derived metrics of age-matched neonates from two sites. The strategy based on the same travelers across different sites may overcome this limitation. However, it is not practical to acquire data from different sites on the same neonates during the same period. Thirdly, the harmonization method is performed on the DTI-derived metrics (FA and MD) instead of the raw diffusion-weighted data. This will increase the cost of training for various metrics.

## Conclusions

In conclusion, the proposed dual GANs-based harmonization method is effective to harmonize neonatal DTI-derived metrics from different sites. Results in this study further suggest that the GANs-based harmonization is a feasible pre-processing method for pooling analyses in multi-center studies. Our future work will focus on the harmonization approach for the raw diffusion-weighted data and try to improve the computational efficiency.

## Methods

This study is approved by the local institutional review board. Informed written consents have been obtained from parents of neonates.

### Pipeline of the GANs-based harmonization method

#### GANs

GANs have achieved impressive results in the image generation, the image editing and the image translation tasks [19, 20]. The main idea of the adversarial training is introducing an auxiliary discriminator to handle the difficulty of evaluating the quality of generated images. The discriminator can be regarded as a binary classifier to distinguish synthetic images from real images. During the training process, the generator tries to generate high-quality synthetic images to satisfy the discriminator, while the discriminator tries to discriminate those synthetic images. In practice, the generator and the discriminator are usually implemented as two independent neural networks. To form the adversarial relationship, GANs use the cross-entropy to define the objective of both networks:

1 $$\mathop {{\text{min}}}\limits_{G} \mathop {\max }\limits_{D}   V\left( {D,G} \right) = {\mathbb{E}}_{{x\sim p_{\text{data}} \left( x \right)}} \left[ {\log D\left( x \right)} \right] + {\mathbb{E}}_{{z\sim p_{z} \left( z \right)}} \left[ {\log \left( {1 - D\left( {G\left( z \right)} \right)} \right)} \right], $$

where *G* and *D* denote the generator and the discriminator separately. $x\sim p_{\text{data}} \left( x \right)$ represents the real data. $z\sim p_{z} \left( z \right)$ is the random noise taken by the generator. The min and max denote that two networks are going to optimize this objective function in opposite directions. The capacity of the generator and the discriminator will be improved during the training process. Once the generator is well trained, it can generate high-quality images, hard to be distinguished from real images.

Based on dual GANs, the harmonization pipeline is designed as shown in Fig. 1. Details about the dual learning architecture, the objective, and the network configuration are introduced as follows.

#### Dual learning architecture

The dual learning architecture is firstly proposed to reduce the requirement on labeled pairs in the machine translation [24]. The main idea of such architecture is to avoid the need of the paired training data. Moreover, the dual learning architecture can also help constraining a one-to-one mapping between the source and the target domains [21].

As shown in Fig. 1, the workflow of the dual GANs-based harmonization can be briefly summarized as follows: given the metric image (such as the FA) $x \in p_{{x\sim {\text{site}}1}} \left( x \right)$, generator $G_{A} :{\text{site }}1 \to {\text{site }}2$ is employed to generate the harmonized output $x^{\prime} = G_{A} \left( x \right)$. Discriminator D_A_ is then trained to distinguish the harmonized result *x*ʹ from the DTI-derived metric $y \in {\text{site }}2$. To guarantee a meaningful mapping, generator $G_{B} :{\text{site }}2 \to {\text{site }}1$ is used to generate the corresponding reconstructed metric $x^{\prime\prime} = G_{B} \left( {x^{\prime}} \right) = G_{B} \left( {G_{A} \left( x \right)} \right)$ of the original input *x*. And a reconstruction loss $\left\| {x - G_{B} (G_{A} \left( x \right))} \right\|$ is employed to force the reconstructed result *x*” to obey the original distribution. Notice that, the generator and the discriminator are trained simultaneously in this dual task. Similarly, the metric $y \in p_{{x\sim {\text{site}}2}} \left( y \right)$ is used to generate the harmonized result $y^{\prime} = G_{B} \left( y \right)$. And then the reconstructed metric is generated by: $y^{\prime\prime} = G_{A} \left( {y^{\prime}} \right) = G_{A} \left( {G_{B} \left( y \right)} \right)$. The reconstruction error is defined as $\left\| {y - G_{A} (G_{B} \left( y \right))} \right\|$, the distance between the original and the reconstructed metrics.

A previous study [21] showed that the conventional model cannot guarantee the one-to-one mapping, since the ability of the generator is theoretically infinite without the dual learning architecture [19, 20]. In other words, there are quantities of mappings between two domains. Though generators can always find a mapping without any constraint, the mapping is not one-to-one. Such harmonization could not bring us the meaningful relationship. Therefore, the dual learning architecture is essential for the GANs-based harmonization.

#### Objective

The key to the great performance of GANs is the use of the adversarial loss between the generator and the discriminator. However, it is difficult to achieve the balance between the generator and the discriminator. As observed in the previous work [25], the failure of GANs’ training is associated with the traditional format loss function based on the optimization toward the Kullback–Leibler divergence between the real and the generated probability. When there is little or no overlap between them, especially at the early training stage, the gradient from the discriminator will vanish and the training will stall. The Wasserstein distance is continuous and provides a usable gradient, which makes the training process more stable. Thus, we employ the loss function based on the Wasserstein distance. The corresponding adversarial loss function is defined as:

2 $$ L_{\text{adv}} = {\mathbb{E}}_{{y\sim p_{\text{site 2}} \left( y \right)}} \left[ {D_{A} \left( y \right)} \right] - {\mathbb{E}}_{{x\sim p_{\text{site 1}} \left( x \right)}} \left[ {D_{A} \left( {G_{A} \left( x \right)} \right)} \right], $$

where $G_{A}$ denotes the generator, $D_{A}$ denotes the discriminator, $x \in p_{{x\sim {\text{site}}1}} \left( x \right)$ and $y \in p_{{x\sim {\text{site}}2}} \left( y \right)$ denote the input metrics from site 1 and site 2, separately.

Different from approximating the Lipschitz continuity based on weighting clips [22], the gradient penalty approach [26] is employed in this work. In practice, the gradient penalty approach can speed up the training process. Thus the adversarial loss becomes:

3 $$ \begin{aligned} L_{\text{adv}} & =  {\mathbb{E}}_{{y\sim p_{\text{site 2}} \left( y \right)}} \left[ {D_{A} \left( y \right)} \right] - {\mathbb{E}}_{{x\sim p_{\text{site 1}} \left( x \right)}} \left[ {D_{A} \left( {G_{A} \left( x \right)} \right)} \right] \\ & \quad - \lambda_{gp} {\mathbb{E}}_{{\hat{x}\sim \left[ {\alpha x + \left( {1 - \alpha } \right)y} \right]}} \left[ {\left( \|{\nabla D_{A} \left( {\hat{x}} \right)\|_{2} - 1} \right)^{2} } \right] \\ \end{aligned} $$

In the above equation, $\hat{x}$ is sampled uniformly along a straight line between a pair of real and generated images. $\lambda_{gp}$ is a constant used to balance function *D*’s outputs and gradient-influenced factors. In our experiments, we set $\lambda_{gp}$ to 10 according to the previous work [26].

The reconstruction loss is also introduced to force translated samples to obey the domain distribution. It has been proved that *L*_2_ distance usually causes blurry results during the image generation [27]. Thus, the reconstruction loss is defined by *L*_1_ distance.

4 $$ L_{\text{recon}} = \|x - G_{B} \left( {G_{A} \left( x \right)} \right)\| + \|y - G_{A} \left( {G_{B} \left( y \right)} \right)\|, $$

where $G_{B} \left( {G_{A} \left( x \right)} \right)$ and $G_{x} \left( {G_{y} \left( y \right)} \right)$ represent the reconstructed metrics. These reconstructed metrics will be similar to original metrics *x* and *y*, when $L_{recon}$ converges to the minimum.

According to experimental results of the previous study [23], removing the adversarial loss substantially degrades the image quality, as does removing the reconstruction loss. Decreases in quantitative measures, such as FCN-scores and the classification performance, also suggest that both the adversarial loss and the reconstruction loss are important to improve the translation quality. Thus, the final loss function is defined as:

5 $$ L = L_{\text{adv}} + \lambda L_{\text{recon}} , $$

where $\lambda$ is a constant used to balance loss functions, because both the adversarial and the reconstruction losses are important to generate high-quality harmonized results. In our experiments, we set $\lambda$ to 20 according to the previous work [22].

Reconstruction loss is designed to preserve the global information. To introduce more local details, the Markovian discriminator is used in this current work (as shown in Fig. 2). With the employment of Markovian discriminators, the feedback from discriminators encourages generators to concentrate on the local information. Thus, the adversarial loss and the reconstruction loss are complementary to each other.

#### Network configuration

The network architecture is shown in Fig. 2. This work uses the identical network architecture for both GANs. U-net is used as the backbone [28]. Generators are configured with the equal number of convolutional and transposed convolutional layers. For networks with 2D-convolutional kernels, the encoder part is composed of convolutional layers with a kernel size of 5 × 5 and stride-2 in the width and the height orientations, followed by a Leaky rectified linear unit (ReLU) function and Batch normalization layers. For networks with 3D-convolutional kernels, the encoder part is composed of convolutional layers with a kernel size of 5 × 5 × 5, followed by a Leaky ReLU function and Batch normalization layers. To combine the low-level information, feature maps from convolutional layers are passed by skip connections, and concatenated with those calculated in corresponding transposed convolutional layers with the identical output size. The combination of information from front layers can help generators to reserve more low-level features. As for the discriminator, we follow the recommendation given in a previous research [26]. All the Batch normalization layers in the discriminator are removed. Thus, discriminators are configured with fully convolutional networks using modules of form convolution layers followed by a Leaky ReLU function.

Harmonization methods based on global-wise parameters are tended to lose local details. The mapping relationships are not identical across different regions of white matter [10]. Thus, we consider the local information through employing a Markovian discriminator. The Markovian discriminator tends to consider local features, compared with the traditional discriminator [22, 23]. It discriminates input images at the patch level rather than the whole image. Differences in structures between the Markovian discriminator and the traditional discriminator are shown in Fig. 2. With a relatively smaller receptive field, such discriminator will concentrate on local details. The overall discriminative output possibility is computed by averaging all responses. Consequently, receiving the feedback from discriminators, generators can be induced to concentrate on the local information. In this study, the patch size is fixed at 30 × 30 for the discriminator with 2D-convolutional kernels and 30 × 30 × 8 for the discriminator with 3D-convolutional kernels, considering the matrix size of DTI-derived metric images. This is different from the size of 70 × 70 employed in previous studies [22, 23].

### Subjects and data acquisition

#### Participants

This study enrolls 84 term neonates without any MRI abnormalities or evidences of any clinical episodes that might cause cerebral damages. As shown in Table 1, the data include DTI on 42 neonates (28 males and 14 females, gestational age range: 37.43–42.00 weeks, median = 40.00 weeks) from site 1 and 42 neonates (28 males and 14 females, gestational age range: 37.14–41.71 weeks, median = 39.71 weeks) from site 2.

**Table 1:** Demographic information of neonates without magnetic resonance imaging abnormalities from two sites

	Site 1 (*n* = 42)	Site 2 (*n* = 42)	*P*
Gestational age (median and range, week)	40.00 (37.43–42.00)	39.71 (37.14–41.71)	0.25
Postmenstrual age (median and range, week)	41.00 (38.29–43.29)	41.07 (38.57–43.71)	0.82
Gender (male:female)	28:14	28:14	1.00
Birth weight (median and range, g)	3300 (1530–4415)	3375 (1250–4170)	0.36

Mann–Whitney U test is used to test the inter-site differences in the gestational age, the postmenstrual age, and the birth weight. Differences in the gender ratio are tested by using the Chi square test

#### MRI acquisition

The acquisition parameters of DTI are listed in Table 2. DTI is performed on two sites by using the same scanner version (General Electric, 3.0 T, Signa HDXT, WI, USA) with the eight-channel head coil. The single-shot spin echo planar imaging sequence is used for the DTI acquisition. DTI protocol in site 1 is carried out with the following parameters: 35 gradient directions; *b* values = 0 and 1000 s/mm^2^; repetition time/echo time = 5500/95 ms; slice thickness = 4 mm without gap; field of view = 180 × 180 mm^2^; and matrix size = 128 × 128. As for the protocol in site 2, DTI is carried out with parameters: 30 gradient directions; repetition time/echo time = 11,000/69.5 ms; b values = 0 and 600 s/mm^2^; slice thickness = 2.5 mm; while the field of view and the matrix size are the same with site 1.

**Table 2:** Acquisition information of diffusion tensor imaging in the sites 1 and 2

	Site 1	Site 2
MRI scanner version	GE Signa HDXT	GE Signa HDXT
Magnetic field	3.0 T	3.0 T
Coil	Eight-channel head coil	Eight-channel head coil
Sequence	Single-shot SE EPI	Single-shot SE EPI
Number of gradient directions	35	30
Number of b_0_	1	8
Nonzero *b* value (s/mm^2^)	1000	600
Repetition time (ms)	5500	11,000
Echo time (ms)	95	69.5
Slice thickness (mm)	4	2.5
Gap (mm)	0	0
Field of view (mm^2^)	180 × 180	180 × 180
Matrix size	128 × 128	128 × 128

*SE* spin echo, *EPI* echo planar imaging

### Data processing

The eddy current correction is performed initially by using the tool in the FMRIB Software Library (FSL) [29]. Brain regions are then extracted by using the Brain Extraction Tool in FSL. Artifact-corrupted images are excluded automatically prior to the tensor estimation [30]. FA and MD maps are calculated by using the FMRIB diffusion toolbox in FSL.

The image registration is performed by using an optimized pipeline [31]. Firstly, the target FA in the native space is selected from subjects in this study. Secondly, images of all the subjects are registered to the target FA by using the combination of the linear and the nonlinear registration. Finally, all individual FA images are normalized to the neonatal FA template [32]. Other DTI-derived metrics (such as MD) are also normalized to the neonatal template space by using the transformation parameter of FA.

To extract DTI-derived metric values along white matter tracts, the tract probabilistic map (cmrm.med.jhmi.edu) is used to determine regions of the left and right CST and the SCC, vulnerable tracts associated with punctuate white matter lesions [33]. FA values are measured at 100 equivalent levels on each tract defined on the atlas [34]. Firstly, images of all subjects are normalized to the neonatal template. Secondly, measurement planes are equally spaced on the tract probabilistic map corresponding to the neonatal template. Measurements are then averaged on each plane. Finally, metrics are measured at 100 equivalent levels. These 100 planes are described as “locations” along the white matter tract in the results section.

### Implementation of different harmonization methods

#### Training procedure of the dual GANs-based method

In this study, the sixfold cross-validation is used for the model training and validation. During the training, we firstly train discriminators and then generators. This work employs the mini-batch Stochastic Gradient Descent and the RMSProp solver. The training process is performed by looping over each training sample until the convergence. In this work, we train the model by 300 epochs to get the loss function converged (Figure S6, Additional file 7). The training takes about 8 h by using a single Nvidia Geforce GTX 1080Ti GPU. To choose the suitable kernel style and the orientation, this study performs the dual GANs by using the 3D-kernel (5 × 5 × 5) and the 2D-kernel (5 × 5) in the axial, coronal and sagittal orientations. To evaluate the feasibility of the dual-GAN method in case of multiple sites, the ComBat method [17] is used to generate the simulated data in a third domain different from site 1 or site 2. In this work, FA maps of the site 3 are the simulated data by transforming the data of site 1 to the third domain. As shown in (Additional file 5: Figure S4A and C), there are significant differences between site 1 and site 3, as well as site 2 and site 3. Data of site 2 is selected as the reference. The training is performed between the other site and the reference.

#### Conventional methods

This study compares the proposed harmonization method with three conventional methods: the global-wise scaling, the voxel-wise scaling, and the ComBat. This work performs these methods based on the description in the previous study [17]. For the global-wise scaling, metric values are averaged in the whole white matter region. Then these averaged values are used to calculate the scaling factor. As a comparison, the voxel-wise scaling calculates the scaling factor at the voxel level. For the ComBat, the harmonization is performed at the voxel level by using the code from https://github.com/Jfortin1/ComBatHarmonization.

### Statistical analysis

Mann–Whitney U test is used to test inter-site differences in the gestational age, the postmenstrual age, and the birth weight. Differences in the gender ratio are tested by using the Chi square test. Tests are considered statistically significant at *P *< 0.05.

To reveal distribution differences in FA of the two different sites, this study calculates the histogram of FA values in the white matter for each subject. The comparison in averaged FA values between sites is performed by using the Mann–Whitney U test. To investigate the correlation between FA values and site effects, Pearson correlation is performed between the inter-site difference and the averaged FA. Inter-site differences are also tested by using the general linear model in TBSS [31]. The permutation number is set at 10,000. Tests in TBSS are considered significant at *P *< 0.05 after the family-wise error rate correction and the threshold-free cluster enhancement. For inter-site comparisons after harmonization, differences with *P *< 0.05 in any run of the cross-validation are overlaid together on the template map. To quantify differences between sites, absolute errors and the RMSE of metric values are also calculated on the white matter before and after harmonization by using different methods.

To investigate age-related alterations before and after harmonization, Pearson correlation is performed between FA along white matter tracts (left CST, right CST and SCC) and the postmenstrual age. To evaluate whether inter-gender differences could be preserved during the harmonization procedure, the effect size (Cohen’s *d* between males and females) is calculated before and after harmonization [7]:

6 $$ d = \frac{{M_{f} - M_{m} }}{{S_{\text{pooled}} }}, $$

where $M_{f}$ and $M_{m}$ are the mean FA of the female and the male subsets separately, $S_{\text{pooled}}$ is the pooled standard deviations for both subsets, which is given by:

7 $$ S_{\text{pooled}} = \sqrt {\frac{{\left( {n_{f} - 1} \right)S_{f}^{2} + \left( {n_{m} - 1} \right)S_{m}^{2} }}{{n_{f} + n_{m} - 2}}} , $$

where $n_{f}$ and $n_{m}$ are the number of females and males separately. $S_{f}$ and $S_{m}$ are the standard deviations for the female and the male groups, respectively.

Performances (including the absolute error, the RMSE, the correlation with age, and the Cohen’s *d*) of harmonization methods are averaged over different runs of the cross-validation.

## Supplementary information

**Additional file 1: Figure S1.** Relationship between inter-site differences and averaged fractional anisotropy (FA) values in the white matter region. *r*: Pearson correlation coefficient.

**Additional file 2: Figure S2.** Inter-site differences in mean diffusivity (MD) and the performance of Dual GANs (axial) on the MD metric. Dual GANs (axial) indicates the harmonization performed by using dual generative adversarial networks with the 2 dimensional kernel on axial slices. For inter-site comparisons after harmonization, differences with *P *< 0.05 in any run of the sixfold cross-validation are overlaid together on the template map. Absolute errors and root mean square errors (RMSE) after harmonization have been averaged over different runs of the sixfold cross-validation.

**Additional file 3: Table S1.** Root mean square error (RMSE) on white matter before and after harmonization by using different methods

**Additional file 4: Figure S3.** Evaluation of the harmonization by Dual GANs (3D) on the fractional anisotropy (FA) metric. Dual GANs (3D) indicates the harmonization performed by using dual generative adversarial networks with the 3 dimensional kernel. For inter-site comparisons after harmonization, differences with *P *< 0.05 in any run of the sixfold cross-validation are overlaid together on the template map. Absolute errors and root mean square errors (RMSE) after harmonization have been averaged over different runs of the sixfold cross-validation.

**Additional file 5: Figure S4.** Evaluation of the harmonization by Dual GANs (axial) on the fractional anisotropy (FA) metric of 3 sites. Dual GANs (axial) indicates the harmonization performed by using dual generative adversarial networks with the 2 dimensional kernel on axial slices. The ComBat method is used to generate the simulated data in a third domain different from site 1 or site 2 (as shown in the following Figure A and C). FA maps of the site 3 are the simulated data by transforming the data of site 1 to the third domain. For inter-site comparisons after harmonization, differences with *P *< 0.05 in any run of the sixfold cross-validation are overlaid together on the template map.

**Additional file 6: Figure S5.** The effect size between genders (Cohen’s *d* between males and females) in site 1 and 2 before and after harmonization. The Cohen’s *d* values after harmonization have been averaged over different runs of the sixfold cross-validation.

**Additional file 7: Figure S6.** Changes of the reconstruction loss (left) and the negative adversarial loss (right) with epochs.

## Data Availability

The codes and datasets of this study are available from the corresponding authors upon reasonable request.

## Abbreviations

CST: corticospinal tract
DTI: diffusion tensor imaging
FA: fractional anisotropy
FSL: FMRIB software library
GANs: generative adversarial networks
MRI: magnetic resonance imaging
ReLU: rectified linear unit
RISH: rotation-invariant spherical harmonic
RMSE: root mean square error
SCC: splenium of the corpus callosum
TBSS: tract-based spatial statistics
2D: 2-dimensional
3D: 3-dimensional
