# Supplementary material for: Inter-site harmonization based on dual generative adversarial networks for diffusion tensor imaging: application to neonatal white matter development
Source: Biomed Eng Online. 2020 Jan 15;19:4. doi: 10.1186/s12938-020-0748-9 (PMC6964111; doi:10.1186/s12938-020-0748-9)

Figure S1. Relationship between inter-site differences and averaged fractional anisotropy (FA) values in the white matter region. *r*: Pearson correlation coefficient.

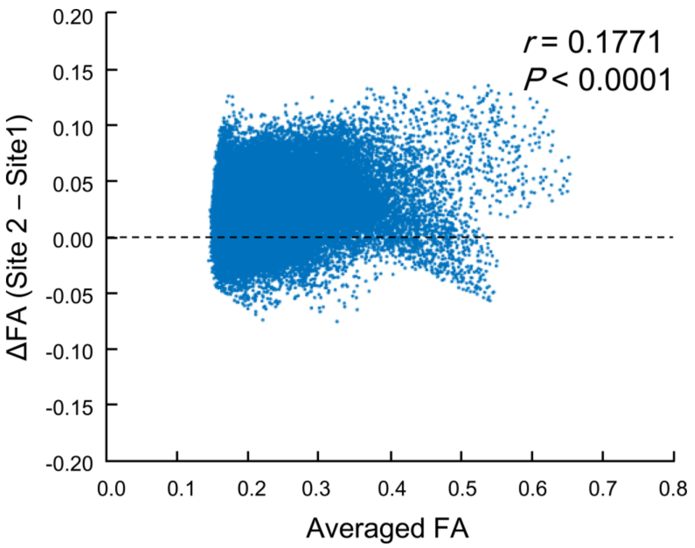

Supplement: Supplementary file 1 — Additional file 1: Figure S1. Relationship between inter-site differences and averaged fractional anisotropy (FA) values in the white matter region. r: Pearson correlation coefficient. [file 12938_2020_748_MOESM1_ESM.pdf]
