# Supplementary material for: Inter-site harmonization based on dual generative adversarial networks for diffusion tensor imaging: application to neonatal white matter development
Source: Biomed Eng Online. 2020 Jan 15;19:4. doi: 10.1186/s12938-020-0748-9 (PMC6964111; doi:10.1186/s12938-020-0748-9)
Supplement: Supplementary file 3 — Additional file 3: Table S1. Root mean square error (RMSE) on white matter before and after harmonization by using different methods [file 12938_2020_748_MOESM3_ESM.pdf]

Table S1. Root mean square error (RMSE) on white matter before and after harmonization by using different methods.

Methods	Reference: Site 1	Reference: Site 2
Original	0.0606	0.0606
Global-wise scaling	0.0646	0.0529
Voxel-wise scaling	0.0542	0.0479
ComBat	0.0508	0.0508
Dual GANs (axial)	0.0470	**0.0391**
Dual GANs (sagittal)	0.0540	0.0497
Dual GANs (coronal)	0.0535	0.0506
Dual GANs (3D)	**0.0408**	0.0409

Note: The values are the medians across subjects. RMSE values after harmonization have been averaged over different runs of the sixfold cross-validation. Bold indicates the smallest RMSE in the column. Dual GANs (axial), Dual GANs (sagittal), and Dual GANs (coronal) indicate the harmonization performed by using dual generative adversarial networks with the 2 dimensional kernel on axial slices, sagittal slices, and coronal slices, respectively. Dual GANs (3D) indicates the harmonization performed by using dual generative adversarial networks with the 3 dimensional kernel.
