# Supplementary material for: Inter-site harmonization based on dual generative adversarial networks for diffusion tensor imaging: application to neonatal white matter development
Source: Biomed Eng Online. 2020 Jan 15;19:4. doi: 10.1186/s12938-020-0748-9 (PMC6964111; doi:10.1186/s12938-020-0748-9)

Figure S3. Evaluation of the harmonization by Dual GANs (3D) on the fractional anisotropy (FA) metric. Dual GANs (3D) indicates the harmonization performed by using dual generative adversarial networks with the 3 dimensional kernel. For inter-site comparisons after harmonization, differences with *P*<0.05 in any run of the sixfold cross-validation are overlaid together on the template map. Absolute errors and root mean square errors (RMSE) after harmonization have been averaged over different runs of the sixfold cross-validation.

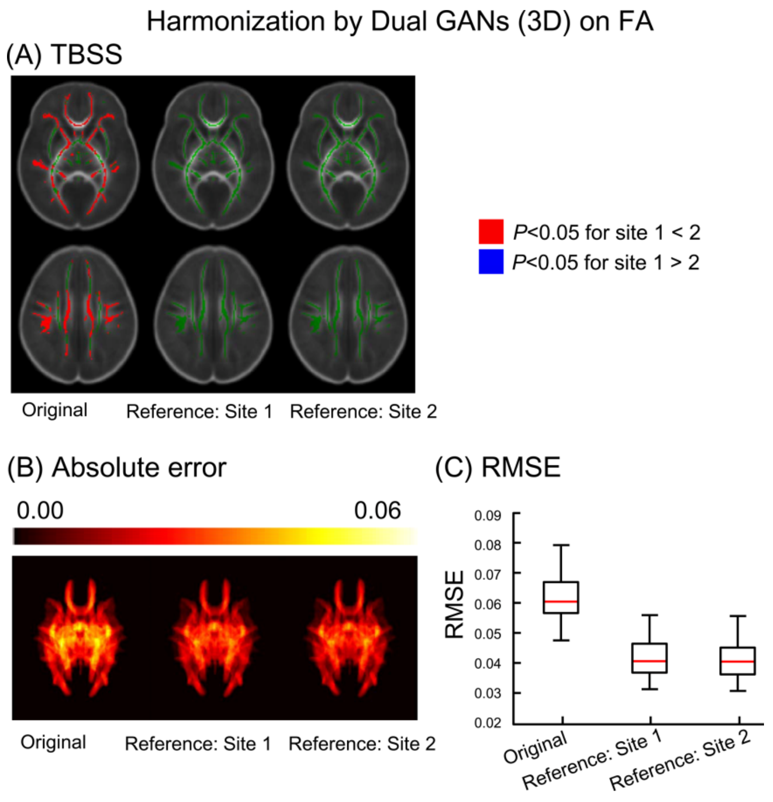

Supplement: Supplementary file 4 — Additional file 4: Figure S3. Evaluation of the harmonization by Dual GANs (3D) on the fractional anisotropy (FA) metric. Dual GANs (3D) indicates the harmonization performed by using dual generative adversarial networks with the 3 dimensional kernel. For inter-site comparisons after harmonization, differences with P < 0.05 in any run of the sixfold cross-validation are overlaid together on the template map. Absolute errors and root mean square errors (RMSE) after harmonization have been averaged over different runs of the sixfold cross-validation. [file 12938_2020_748_MOESM4_ESM.pdf]
