# Supplementary material for: Inter-site harmonization based on dual generative adversarial networks for diffusion tensor imaging: application to neonatal white matter development
Source: Biomed Eng Online. 2020 Jan 15;19:4. doi: 10.1186/s12938-020-0748-9 (PMC6964111; doi:10.1186/s12938-020-0748-9)

Figure S4. Evaluation of the harmonization by Dual GANs (axial) on the fractional anisotropy (FA) metric of 3 sites. Dual GANs (axial) indicates the harmonization performed by using dual generative adversarial networks with the 2 dimensional kernel on axial slices. The ComBat method is used to generate the simulated data in a third domain different from site 1 or site 2 (as shown in the following Figure A and C). FA maps of the site 3 are the simulated data by transforming the data of site 1 to the third domain. For inter-site comparisons after harmonization, differences with *P*<0.05 in any run of the sixfold cross-validation are overlaid together on the template map.

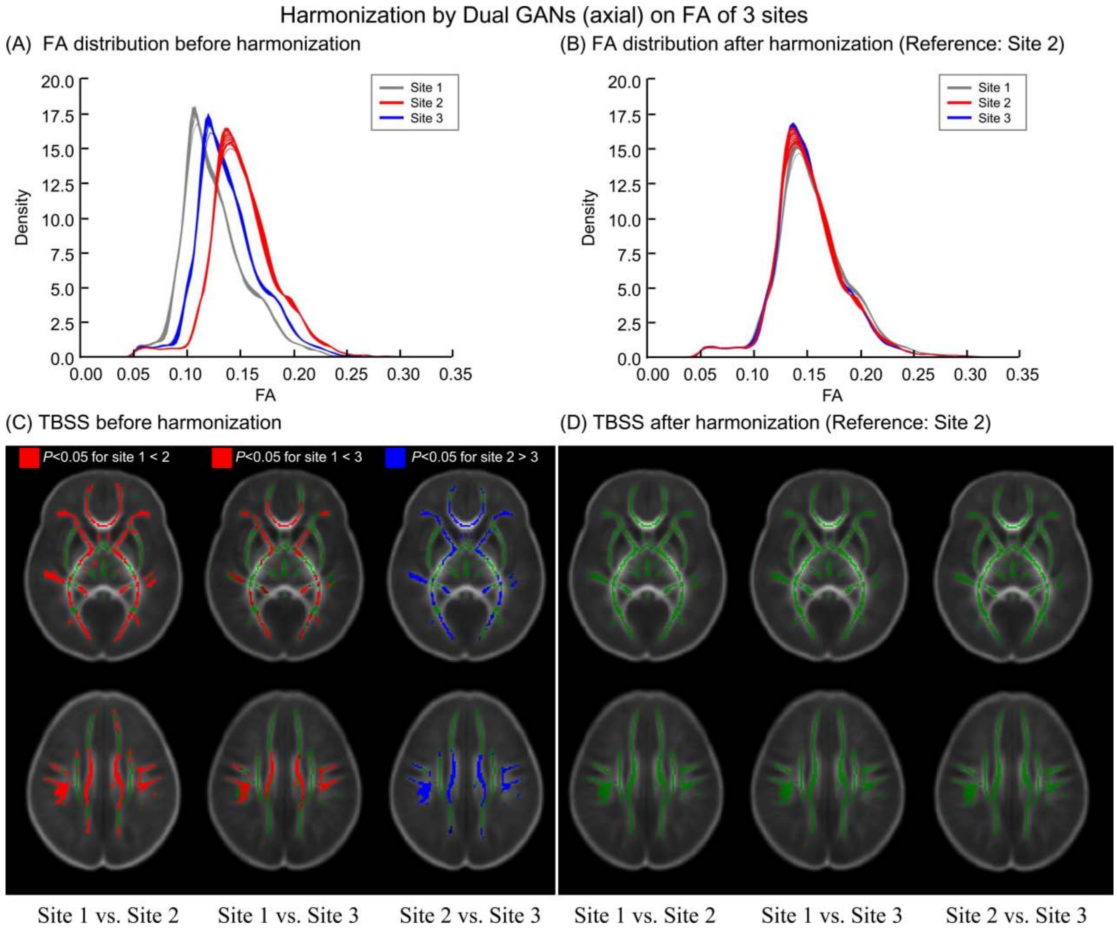

Supplement: Supplementary file 5 — Additional file 5: Figure S4. Evaluation of the harmonization by Dual GANs (axial) on the fractional anisotropy (FA) metric of 3 sites. Dual GANs (axial) indicates the harmonization performed by using dual generative adversarial networks with the 2 dimensional kernel on axial slices. The ComBat method is used to generate the simulated data in a third domain different from site 1 or site 2 (as shown in the following Figure A and C). FA maps of the site 3 are the simulated data by transforming the data of site 1 to the third domain. For inter-site comparisons after harmonization, differences with P < 0.05 in any run of the sixfold cross-validation are overlaid together on the template map. [file 12938_2020_748_MOESM5_ESM.pdf]
