# Supplementary material for: Inter-site harmonization based on dual generative adversarial networks for diffusion tensor imaging: application to neonatal white matter development
Source: Biomed Eng Online. 2020 Jan 15;19:4. doi: 10.1186/s12938-020-0748-9 (PMC6964111; doi:10.1186/s12938-020-0748-9)

Figure S5. The effect size between genders (Cohen's *d* between males and females) in site 1 and 2 before and after harmonization. The Cohen's *d* values after harmonization have been averaged over different runs of the sixfold cross-validation.

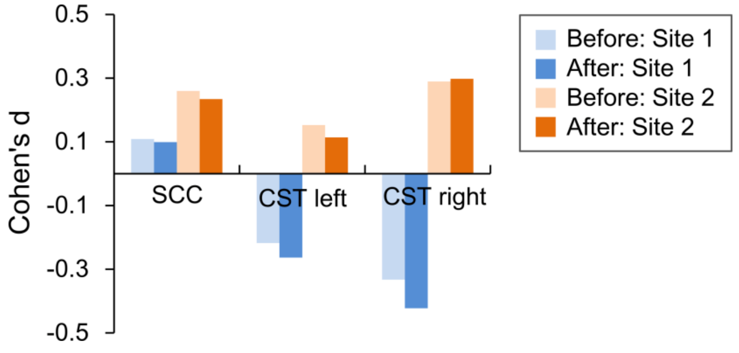

Supplement: Supplementary file 6 — Additional file 6: Figure S5. The effect size between genders (Cohen’s d between males and females) in site 1 and 2 before and after harmonization. The Cohen’s d values after harmonization have been averaged over different runs of the sixfold cross-validation. [file 12938_2020_748_MOESM6_ESM.pdf]
