# Supplementary material for: Inter-site harmonization based on dual generative adversarial networks for diffusion tensor imaging: application to neonatal white matter development
Source: Biomed Eng Online. 2020 Jan 15;19:4. doi: 10.1186/s12938-020-0748-9 (PMC6964111; doi:10.1186/s12938-020-0748-9)

Figure S6. Changes of the reconstruction loss (left) and the negative adversarial loss (right) with epochs.

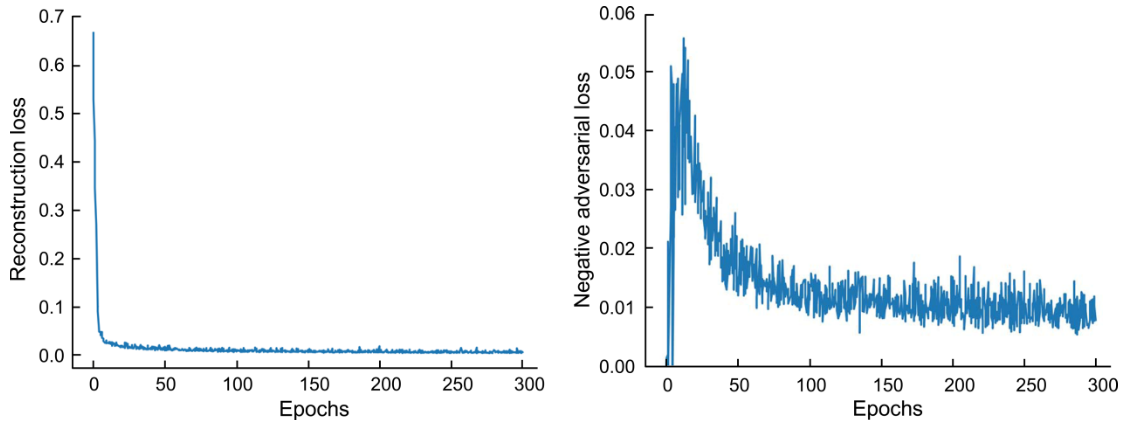

Supplement: Supplementary file 7 — Additional file 7: Figure S6. Changes of the reconstruction loss (left) and the negative adversarial loss (right) with epochs. [file 12938_2020_748_MOESM7_ESM.pdf]
